# Taking the common into account: phylogeographical patterns of native, non-endemic plant species of the Canary Islands

**DOI:** 10.1093/aobpla/plag021

**Published:** 2026-05-20

**Authors:** Carlos García-Verdugo, Mario Mairal, Ana Martínez, Fouad Msanda, Cherif Harrouni, Julio Peñas, Juli Caujapé-Castells

**Affiliations:** Departamento de Botánica, Universidad de Granada, Av. de Fuentenueva s/n, 18071 Granada, Spain; Departamento de Biodiversidad, Ecología y Evolución, Universidad Complutense de Madrid, c/ José Antonio Novais 12, 28040 Madrid, Spain; Departamento de Biodiversidad, Ecología y Evolución, Universidad Complutense de Madrid, c/ José Antonio Novais 12, 28040 Madrid, Spain; Laboratory of Biotechnology and Natural Resources Valorization, Faculty of Sciences of Agadir, Ibn Zohr University, BP 8106 Agadir, Morocco; Hassan II Institute of Agronomy and Veterinary Sciences, km 2 route de Taroudant, BP 121 Agadir, Morocco; Departamento de Botánica, Universidad de Granada, Av. de Fuentenueva s/n, 18071 Granada, Spain; Departamento de Biodiversidad Molecular y Banco de ADN, Jardín Botánico Canario ‘Viera y Clavijo’—Unidad Asociada CSIC, Cabildo de Gran Canaria, Camino del Palmeral 15, 35017 Las Palmas de Gran Canaria, Spain

**Keywords:** genetic diversity, island colonization, *Launaea arborescens*, *Lycium intricatum*, native nonendemics, plastid DNA, stepping-stone differentiation

## Abstract

Islands sustain an outstanding proportion of Earth’s biodiversity. However, modern molecular studies on oceanic island floras have typically focused on endemics, which leaves many research questions involving other types of taxa unaddressed. In this study, we analyse the patterns of genetic variation in two co-occurring native nonendemics (NNE) that are common components of lowland habitats across the Canarian archipelago: *Launaea arborescens* (Asteraceae), a wind-dispersed species, and *Lycium intricatum* (Solanaceae), an endozoochorus species. We predicted that, given their presumably recent colonization of the archipelago, both lineages should show a genetic pattern compatible with a stepping-stone model, i.e. from the easternmost islands (closest to mainland Africa) to the western islands. We carried out exhaustive sampling in all the islands of distribution and in neighbouring mainland areas (SW Morocco). Using three plastid DNA regions, we calculated levels of haplotype diversity, conducted analyses of spatial distribution of molecular variance, and tested various models of colonization and gene flow with complementary coalescent-based approaches. For comparison, we additionally performed a literature review to assess general patterns of haplotype diversity on plant species with similarly widespread distributions across the archipelago. A stepping-stone model of island colonization was only partly supported by our results, since back-colonization of mainland areas was a scenario equally supported by coalescent analyses and estimates of historical gene flow. Both species showed that the easternmost islands represented *de facto* a genetic continuum of the neighbouring mainland area. However, *Lycium* generally displayed much higher rates of gene flow than *Launaea*, which may be due to secondary dispersal mediated by predatory birds. Lastly, our literature survey revealed that both NNE harbour unprecedented high levels of genetic diversity on the easternmost Canary islands. This study illustrates that the phylogeographical analysis of NNE provides novel insights into island biogeography, since these taxa can deviate from common patterns observed in endemic lineages.

## Teaser

Islands sustain an outstanding proportion of Earth’s biodiversity, but native non-endemic (NNE) species have been seldom analyzed in phylogeographical studies. Here, we analyze the patterns of genetic variation in two co-occurring NNE that are common components of lowland habitats across the Canarian archipelago: Launaea arborescens (Asteraceae), a wind-dispersed species, and Lycium intricatum (Solanaceae), an endozoochorus species. Both species showed that the easternmost islands represented de facto a genetic continuum of the neighbouring mainland area, and frequent events of back-colonization of mainland from the islands was supported by our analyses. A literature survey revealed that both NNE harbour unprecedented high levels of genetic diversity on the easternmost Canary islands. This study illustrates that the phylogeographical analysis of NNE provides novel insights into island biogeography, since these taxa can deviate from common patterns observed in endemic lineages.

## Introduction

Island biodiversity has fascinated researchers for centuries, and much of this interest can be explained by some outstanding figures: while islands represent a small (c. 7%) fraction of the Earth’s emerging land, they sustain c. 20% of its biota (Fernández-Palacios *et al.* 2021a). However, when it comes to modern studies in island plants, molecular research has strongly focused on endemics, as they represent excellent study cases to address evolutionary, biogeographical and conservation issues (Patiño *et al.* 2017, Cerca *et al.* 2023, Schrader *et al.* 2024). Thus, although the term ‘island’ is intrinsically associated with peculiar biodiversity at all levels (including genetic variation), native non-endemics (NNE, hereafter) have been seldom considered in phylogeographical studies (for some exceptions, see Chiang *et al.* 2006, Caetano *et al.* 2012). Such a bias is remarkable, since these taxa represent a major fraction of every island biota (Schrader *et al.* 2024), and therefore information on the spatial distribution of their genetic diversity can help us understand key processes in island biology, such as community assembly or colonization patterns (Patiño *et al.* 2017).

Despite current trends in scientific research, we argue that there are several reasons why the questions addressed by phylogeographical studies in island endemics should be expanded to NNE taxa. First, they may help understand processes related to recent island colonization, including incipient local adaptation to new environments or the effects of genetic drift on population differentiation (Caetano *et al.* 2012, Yamada and Maki 2012, Yoichi *et al.* 2021). In other words, since a large proportion of island endemics (i.e. excluding those originated through *in situ* radiation) were once native non-endemics, the latter may provide key insights into the process of speciation. Second, some phylogeographical studies have shown that species listed in island biodiversity inventories as NNE actually represent genetic lineages that are strongly differentiated from their mainland counterparts (Coello *et al.* 2021, García-Verdugo *et al.* 2021, Kimura *et al.* 2022), which calls for taxonomic revision and, ultimately, reassessment of biodiversity catalogues. In addition, NNE are often key functional components of island habitats (‘ecological winners’, *sensu* Fernández-Palacios *et al.* 2021b), and inferring patterns of molecular variation in NNE would thus prove useful for conservation and restoration programmes (Hoban *et al.* 2021).

In this study, we analyse the patterns of genetic variation in two widespread NNE taxa of the Canary Islands. More than 50% of the native flora of this archipelago is composed of NNE (for a recent floristic assessment, see Beierkuhnlein *et al.* 2021), which is also one of the most prominent biogeographical features of the Macaronesian flora (Fernández-Palacios *et al.* 2024). Previous studies have highlighted that contrasting distribution patterns between Canarian endemic and NNE plants exist: while a substantial proportion of endemics are currently absent on the easternmost islands of Lanzarote and Fuerteventura (which are also the oldest and closest to mainland Africa), they harbour most NNE (Bramwell 1976, Reyes-Betancort *et al.* 2008). Since colonization of the easternmost Canarian islands (ECI, hereafter) from the mainland is the most likely step for subsequent dispersal across the archipelago, the lack of widespread endemics on ECI could be explained by local extinction (i.e. island extirpation), which may have been more intense throughout late-Pleistocene environmental shifts (Fernández-Palacios *et al.* 2016, García-Verdugo *et al.* 2019a, Caujapé-Castells *et al.* 2022). The recent colonization of the ECI inferred for some Canarian endemics either by migration from the central islands or from mainland Africa supports this possibility (García-Verdugo *et al.* 2019a, Albaladejo *et al.* 2021, Vitales *et al.* 2023, Coello *et al*. 2024b, Rincón Barrado *et al.* 2024). Another consequence of island extirpation on the ECI is that one of the expectations of the ‘progression rule’, i.e. sequential dispersal from older (eastern) to younger (western) islands (Shaw and Gillespie 2016), is not recovered in most phylogeographical reconstructions of endemic lineages (for recent examples, see White *et al.* 2020, Coello *et al*. 2024b, Rincón Barrado *et al.* 2024). If we assume that, unlike most endemics, NNE taxa are recent colonizers not affected by Pleistocene extirpation, it follows that we could expect for these taxa a pattern of colonization consistent with east-to-west sequential dispersal (i.e. following a stepping-stone model; García-Verdugo *et al.* 2019a). Testing this hypothesis requires the analysis of NNE with widespread distributions within the archipelago, which would also provide a complementary view of the spatial patterns of genetic diversity with regard to that documented in narrow endemics (Romeiras *et al.* 2015, Martín-Hernanz *et al.* 2019). In the case of the Canarian archipelago, lowland xerophytic taxa are exceptional candidates for hypothesis testing, since that type of habitat is found on all major islands (del Arco and Rodríguez-Delgado 2018).

Our study focused on two NNE that show contrasting fruit morphologies that may be related to different modes of dispersal. Indeed, dispersal mechanisms are thought to play a fundamental role in the spatial extent of archipelago colonization (Arjona *et al.* 2018). For instance, some molecular studies on Canarian lineages suggest that fruits bearing plumose structures suited for wind-dispersal promote extensive within-island gene flow, but this may be a poor mechanism for among-island dispersal (Jones *et al.* 2014, García-Verdugo *et al.* 2017, 2019b). Fleshy-fruited species, in contrast, often show a weak genetic structure within archipelagos (García-Verdugo et al. 2015; Betzin *et al.* 2016). We could therefore expect fleshy-fruited (particularly, endozoochorous) species to show weaker genetic structure across the archipelago than that displayed by wind-dispersed species (García-Verdugo *et al.* 2015, Arjona *et al.* 2018). In agreement with this expectation, the syngameon hypothesis (Caujapé-Castells *et al.* 2017) predicts that bidirectional dispersal between mainland source areas and the Canary Islands may be promoted in species with high dispersal ability, given the limited geographical isolation between both regions (less than 90 km in some areas). As a result, extensive gene flow would enhance genetic diversity at local scales, particularly on the ECI (Fernández-Palacios *et al.* 2016, Caujapé-Castells *et al.* 2017, 2022). Studies thus far have revealed limited levels of genetic diversity in plant endemics occurring on ECI (e.g. Coello *et al.* 2024a, 2024b, González Carracedo *et al.* 2024), although little is known on whether this pattern is also applicable to native, recent colonizers presumably less impacted by Pleistocene extinction.

Based on these premises, our objectives are: (i) to test whether two co-occurring NNE display similar spatio-temporal patterns of island colonization, which are expected to be compatible with a stepping-stone model, and (ii) to investigate if the spatial pattern of genetic variation shown by our focal species is similar to that described in other widespread species of the Canarian archipelago. With this study, we aim to gain some insight into the biogeographical patterns of two key NNE taxa of lowland areas of the Canary Islands, but also seek to stimulate further phylogeographical research on one fraction of island biodiversity that has been poorly investigated to date.

## Materials and methods

### Study species

*Launaea arborescens* (Batt.) Murb. (Asteraceae) and *Lycium intricatum* Boiss. (Solanaceae) are two species with largely overlapping distributions across the western Mediterranean basin, Atlantic areas of NW Africa (Morocco and Mauritania) and several Macaronesian islands (GBIF 2025). Both species display a combination of traits (i.e. strong resprouting ability, spiny twigs and small, drought-deciduous leaves) that favours colonization of xerophytic habitats (García-Verdugo *et al.* 2024); hence, they typically coexist as predominant components of lowland areas (up to 500 m a.s.l.) on the central and eastern islands of the Canarian archipelago (del Arco and Rodríguez-Delgado 2018). Remarkably, they are absent (*Lycium*) or extremely rare (*Launaea*) on the westernmost islands of La Palma and El Hierro (Padrón-Mederos and León 2010), which suggests that both species may be still undergoing a process of range expansion within the archipelago.

Considering their fruit morphology, each species displays a different dispersal syndrome. *Launaea arborescens* is an anemochorous species (Vargas *et al.* 2023), as its fruits are achenes attached to a plumose pappus that promotes dispersal by wind (Fig. 2a). However, when compared to other anemochorous species, it shows average levels of potential dispersal ability (as estimated by terminal velocity) (García-Verdugo *et al.* 2019b). In contrast, *L. intricatum* is an endozoochorous species (Vargas *et al.* 2023) which produces reddish, small (5–10 mm) berries that are dispersed internally by animals (Fig. 2b). Although their morphological fruit traits are clearly associated with these dispersal vectors, it has been suggested that secondary dispersal by birds preying on frugivorous lizards of the genus *Gallotia* may also play a role in both species (Padilla *et al.* 2012).

### Population sampling

Sampling was performed to represent the general distribution of our focal species in the Canarian archipelago. Since the ECI (Lanzarote and Fuerteventura) are a fundamental area for subsequent archipelago colonization, and both species are very common on these two islands, they were represented with a minimum of six populations. For the western and central islands (Gran Canaria, Tenerife and La Gomera; WCI hereafter), population sampling was roughly proportional to the occurrence of the species on each island (2–4 populations, depending on island size). Additionally, four populations of each species were sampled in mainland SW Morocco (M, hereafter) to assess patterns of gene flow and compare levels of genetic diversity between the islands and the neighbouring continental area. In total, 21 *Lycium* and 20 *Launaea* populations were considered for this study (Supplementary Table S1). Individuals were sampled at a minimum distance of 20 m from one another at the core of each population. Vouchers for at least one population from each island and the mainland area were deposited at the herbarium of Jardín de Aclimatación de la Orotava (Tenerife, Spain; *Launaea*: ORT 46475–46483, and *Lycium*: ORT 46448–46458).

### DNA sequencing

Leaves from five to six individuals per population were used for genomic DNA extraction following previous studies (García-Verdugo *et al.* 2017, 2019b). For *Launaea* samples, DNA extraction was preceded by the washing pretreatment described in Sun *et al.* (2009). In order to screen the levels of plastid DNA polymorphism in the study species, a set of eight markers described in Shaw *et al.* (2007) was tested using a subset of mainland and island samples. Following the results of this test, the three most polymorphic regions in each species were chosen to extend DNA amplification to the total sample. For *Lycium,* we amplified the plastid regions *psbJ–petA*, 3′*rps16*–5′*trnK* and *ndhF–rpl32* (2339 bp in total, sample size = 121 individuals). For *Launaea*, we amplified the plastid regions 3′*trnV–ndhC*, 3′*rps16*–5′*trnK* and *rpl32–trnL* (1539 bp in total, sample size = 118 individuals). PCR amplification was carried out in a total volume of 25 µl following the conditions detailed in Shaw *et al.* (2007). Data from the three plastid regions were concatenated for each species using DnaSP v5 (Librado and Rozas 2009) and the resulting matrices were used for all subsequent analyses (see Data availability statement).

### Spatial distribution of genetic diversity

To investigate the spatial distribution of genetic diversity across the study area, we first calculated levels of haplotype diversity (*H_d_*) at the population level using DnaSP v5 (Librado and Rozas 2009). Haplotype diversity was then compared among geographical groups (i.e. mainland, ECI, and WCI) using the *MultNonParam* package in R (R Core Team 2023). Haplotype relatedness was examined in each species by constructing a median joining network (Bandelt *et al.* 1999) with the software NETWORK 10.2 (http://www.fluxus-engineering.com).

In addition, we examined the distribution of genetic variation among hierarchical levels (geographical groups and populations) with analyses of molecular variance (AMOVA; Excoffier and Lischer 2010) implemented in the R package *strataG* (Archer *et al.* 2017). To consider the spatial location of populations, we also performed spatial analyses of molecular variance (SAMOVA; Dupanloup *et al.* 2002) using the *Launaea* and *Lycium* datasets separately.

### Inference of colonization models and gene flow patterns

We first explored phylogenetic relationships among samples using the concatenated alignment of the three plastid loci for each species in MrBayes (Ronquist *et al.* 2012). Markov chain Monte Carlo analyses were run for 2 000 000 generations under a GTR substitution model, with two independent runs, each consisting of four chains. Trees were sampled every 1000 generations, and the first 25% of samples were discarded as burn-in. A majority-rule consensus tree was used to summarize posterior probabilities of clades.

**Figure 1:**
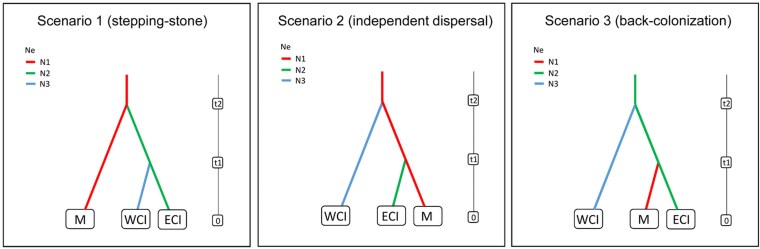
Models of colonization tested for *Lycium* and *Launaea* using plastid DNA sequence data in the coalescent framework implemented in DIYABC-random forest. Populations were geographically grouped into M (mainland area), ECI (easternmost Canary Islands), and WCI (Western Canary Islands). *N* (effective population size) and *t* (divergence time) indicate parameters of interest for demographic inference.

Because the results based on phylogenetic inference showed poor resolution (i.e. most nodes were weakly supported and island and mainland samples were intermingled; see Supplementary Figure S1), we inferred models of colonization and gene flow following coalescent models. First, we used the approximate Bayesian computation (ABC) approach implemented in DIYABC-Random Forest (Collin *et al.* 2021), which is an extended version of the coalescence simulation of DIYABC (Cornuet *et al*. 2014). In-depth phylogeographical studies have revealed that patterns of archipelago colonization may be complex, as they are often driven by local extinction, multiple introductions from mainland or back-colonization of mainland areas from insular populations (Igea *et al.* 2015, Caujapé-Castells *et al.* 2017; García-Verdugo *et al.* 2019b). With the aim of minimizing computation complexity, we grouped populations into three main clusters: mainland (M), Lanzarote and Fuerteventura (ECI), and Gran Canaria, Tenerife and La Gomera (WCI). Such a grouping allowed us to test three main scenarios of colonization typically reported for the Canarian flora involving the mainland area (reviewed in García-Verdugo *et al.* 2019a; Fig. 1). Scenario 1 represented a stepping-stone model of colonization (i.e. our working hypothesis), in which M is the ancestral group and island populations are sequentially derived from east to west, i.e. ECI is derived from M (at *t*_2_), and WCI is derived from ECI (at *t*_1_). This scenario has been supported by molecular data in widespread endemics with a presumably recent origin (Saro *et al.* 2015, Villa-Machío *et al*. 2020). Scenario 2 represented a case of repeated colonization of the archipelago, in which WCI and ECI are colonized by different dispersal events from M. This scenario fits the genetic pattern shown by lineages likely affected by Pleistocene extirpation on ECI: a second wave of colonization from the mainland would create ECI populations genetically closer to mainland counterparts (García-Verdugo *et al.* 2017, 2023). Scenario 3 represented a case of mainland back-colonization, in which ECI was the source group for WCI colonization (at *t*_2_), but also for a more recent establishment (at *t*_1_) of neighbouring mainland (M) populations. This scenario has been supported in several plant lineages (see e.g. Caujapé-Castells 2004, Durán *et al.* 2020, Jaén-Molina *et al.* 2021), and implies that mainland populations may be the result of a relatively recent dispersal from the islands. In order to select the most realistic scenario supported by our data, we conducted 10 000 simulations per scenario in DIYABC-RF following Pudlo *et al.* (2016). The prior distribution of parameters was set as uniform, and the priors for the divergence times were set in the order established in each coalescent model (*t*_2_ > *t*_1_; Fig. 1). The scenario with the highest posterior probability is typically selected as the most likely (Pudlo *et al*. 2016, Raynal *et al.* 2019), but our results were very similar for two of the tested scenarios (see below). We assessed the quality of the predicted scenarios by the visual representation of a linear discriminant analysis (LDA), in which training and observed datasets are projected onto the first two LDA axes (Collin *et al.* 2021). Because we were interested in exploring the temporal pattern of island colonization, we estimated posterior distribution values of parameters *t*_2_ and *t*_1_ (divergence times; Fig. 1) for the two models receiving the highest number of votes using a regression by RF methodology (Raynal *et al.* 2019). Based on a training set of 100 000 simulations, a classification forest of 10 000 decision trees was used to conduct the parameter estimations. To convert divergence time estimates to years, we relied on the observation that both species typically set fruit within the second year of cultivation under greenhouse conditions (F. Cabrera, personal observation; S. Scholz, personal observation). Since field conditions most likely translate into slower growth rates than those observed under greenhouse conditions, we used a conservative estimate for generation time of 5 years for each species.

Following the results from DIYABC-RF, we aimed to investigate whether gene flow among population groups may have had an impact on the biogeographical pattern recovered for both species. We tested alternative models of historical gene flow using the coalescent approach implemented in the software MIGRATE 4.4.3 (Beerli *et al.* 2019). For each species, the plastid sequence dataset was grouped into the three geographical groups used in previous analyses (i.e. M, ECI, and WCI), and four models were run with each dataset (Supplementary Figure S2). Model 1 was the simplest, and assumed a stepping-stone pattern of differentiation with complete isolation (i.e. lack of gene flow) between geographical groups; model 2 incorporated unidirectional gene flow between geographical groups, whereas model 3 and model 4 considered bidirectional gene flow between M and ECI (model 3) or between each neighbouring group (M-ECI and ECI-WCI; model 4). For each model, one long Markov chain was run saving 50 000 generations with sampling increments of 100 generations after a burn-in period of 1 000 000 generations. The number of concurrent chains (replicates) was set to 10. Prior distributions were uniform with a range from 0 to 0.100, for effective population size (Θ, which is the effective population size multiplied by the mutation rate), and 0–100 for migration rate (M, which is the mutation-scaled immigration rate). The static heating scheme included four temperatures of 1, 1.5, 3.0, and 1 000 000. For the most likely model in each case, estimates of M and Θ were used to calculate the number of migrants per generation (Nm).

### Literature survey

To compare the levels of genetic diversity found in our study species with those previously published in other species with widespread distributions in the Canarian archipelago (i.e. occurring on three or more islands), we performed a literature review. To make results comparable, we focused on studies reporting levels of plastid DNA variation and considering a representative sampling of populations (two or more) on each island where the plant species occur. We searched for different combination of terms in the Web of Science database (Clarivate Analytics), namely ‘Canar* islands’, ‘Canar* archipelago’, ‘Macaronesia’, ‘plant populations’, ‘plastid’, ‘chloroplast’, ‘haplot*’, ‘haplotype network’, ‘haplotype diversity’, ‘cpDNA’, ‘pDNA’, ‘cpSSR’, ‘phylogeogr*’. Our personal databases were also checked to find potential studies of interest that we may have missed using our search strategy. From each study, we extracted the data on haplotype diversity reported at the population level. If only haplotype frequencies were reported, haplotype diversity was calculated following Nei (1987) as:


$$H_{d}=\frac{N}{N-1}\sum_{i}\left(x_{i}^{2}\right)$$

where *N* is the sample size in a given population/island and *x_i_* is the frequency of each haplotype in the sample.

Haplotype diversity was averaged over populations or calculated for a given island when data were not provided at the population level. Because we were particularly interested in the patterns of genetic variation on ECI (see Results), the number of haplotypes on ECI populations for each species was compared with the total number of haplotypes found across the archipelago. To detect potential biases in the genetic estimates retrieved by our bibliographical search, we tested for the correlation between sampling effort (i.e. number of individuals and length of plastid DNA sequence considered) and the haplotype diversity detected in each study (see Coello *et al.* 2021). A nonsignificant correlation between sampling effort and haplotype diversity across studies would suggest that our inferences from the bibliographical survey are not substantially affected by sampling differences.

## Results

### Geographical distribution of genetic diversity

**Figure 2:**
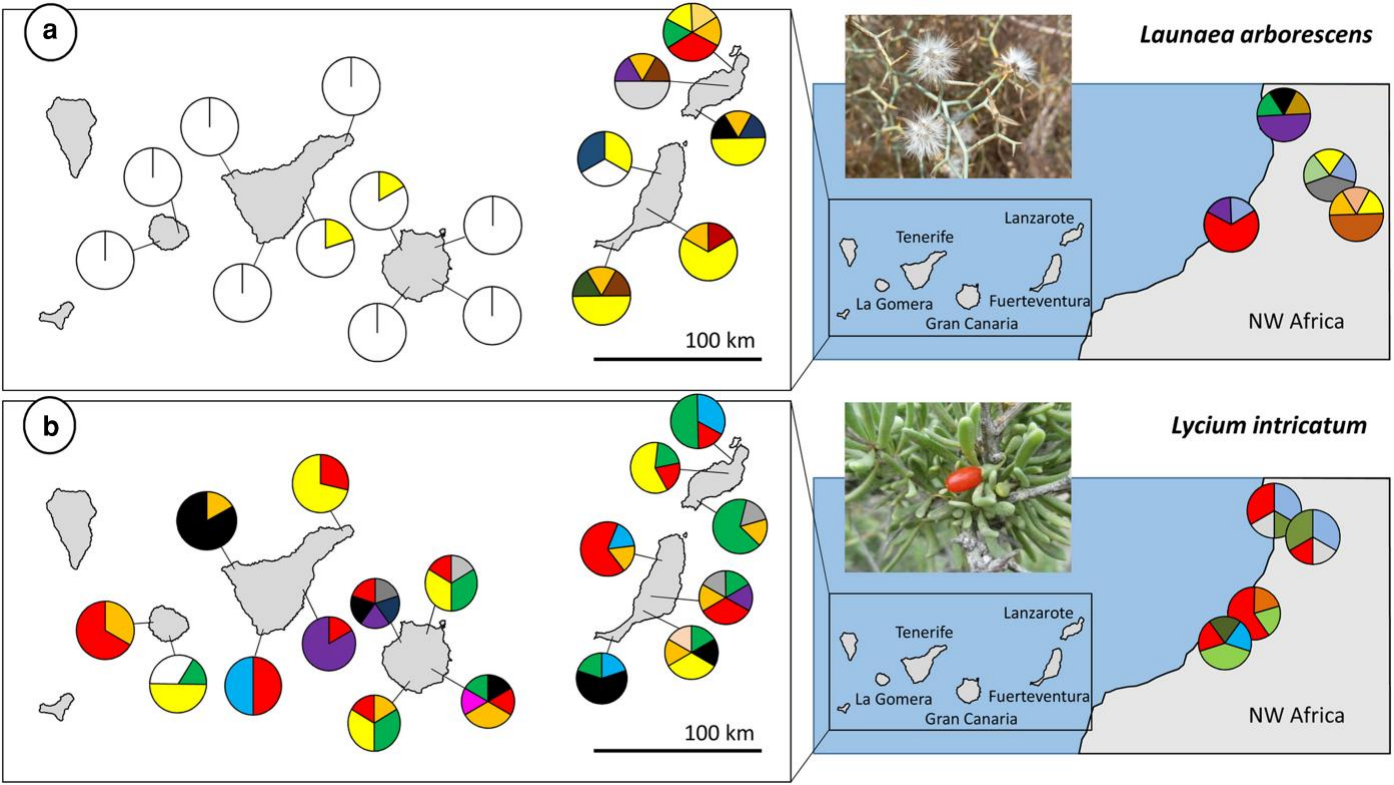
Distribution of haplotypes in island and mainland populations of *Launaea arborescens* (a) and *Lycium intricatum* (b) found in this study. Each colour represents a haplotype and pie charts represent the proportion of each haplotype in a given population. Fruit morphology of each species is shown in the photographs (credit: C. García-Verdugo).

For *Launaea*, plastid DNA sequences revealed 19 haplotypes based on the combination of six nucleotide substitutions and five indels (Supplementary Table S1, Supplementary Figure S3). Six of these haplotypes were only found in mainland populations, seven were only found in island populations, and six were shared between island and mainland populations (Fig. 2, Supplementary Table S1). For *Lycium*, we detected 17 haplotypes based on the combination of six nucleotide substitutions and six indels (Supplementary Table S1, Supplementary Figure S3). Five of these haplotypes were only found in mainland populations, 10 of them were only found in island populations and two were widespread among mainland and island populations (Fig. 2, Supplementary Table S1). The distribution range of island haplotypes was remarkably different between both species. Excluding rare haplotypes (i.e. those found in only one individual of the total sample), 8 out of 10 haplotypes in *Launaea* were restricted to one or two islands. In contrast, 8 out of 9 haplotypes in *Lycium* were found across 3–5 islands (Supplementary Figure S4).

In agreement with the previous pattern, the AMOVA showed that the spatial distribution of genetic diversity in the study area differed between both species. Thus, most of the genetic variation (66.8%) in *Launaea* was found among the three geographical groups (i.e. mainland, ECI and WCI), but *Lycium* did not show significant differentiation among them (‘Among groups’ level only accounted for 1.40% of explained genetic variance; Table 1). Furthermore, genetic differentiation among populations within each group was negligible in *Launaea* (3.30% of explained genetic variation, *P* > .05), but strong in *Lycium* (26.4%; Table 1). Lastly, while both species showed significant levels of genetic variation within populations (*P* < .001 in both cases), this level explained most of the variation found in *Lycium* (72.5%, Table 1). SAMOVA reached an asymptotic value in the percentage of explained variance for *K* = 2 in both species (‘Among groups’ level; Supplementary Table S2). Those two groups included: all mainland + ECI vs. all WCI populations in *Launaea*, and two mainland vs. the rest of populations in *Lycium* (Supplementary Figure S5).

**Table 1:** AMOVA results showing the partition of molecular variance among hierarchical levels in *Launaea arborescens* and *Lycium intricatum* using plastid DNA sequences.

	Among areas		Among pops (areas)		Within pops	
	VC	%Var	VC	%Var	VC	%Var
*Launaea*	7.26***	66.8	0.36^ns^	3.30	3.25***	29.9
*Lycium*	0.04^ns^	1.40	0.73***	26.4	2.00***	72.2

Significance level of variance components: ns= nonsignificant, ***P < .001.

VC, variance component; %Var, percentage of explained variance.

**Figure 3:**
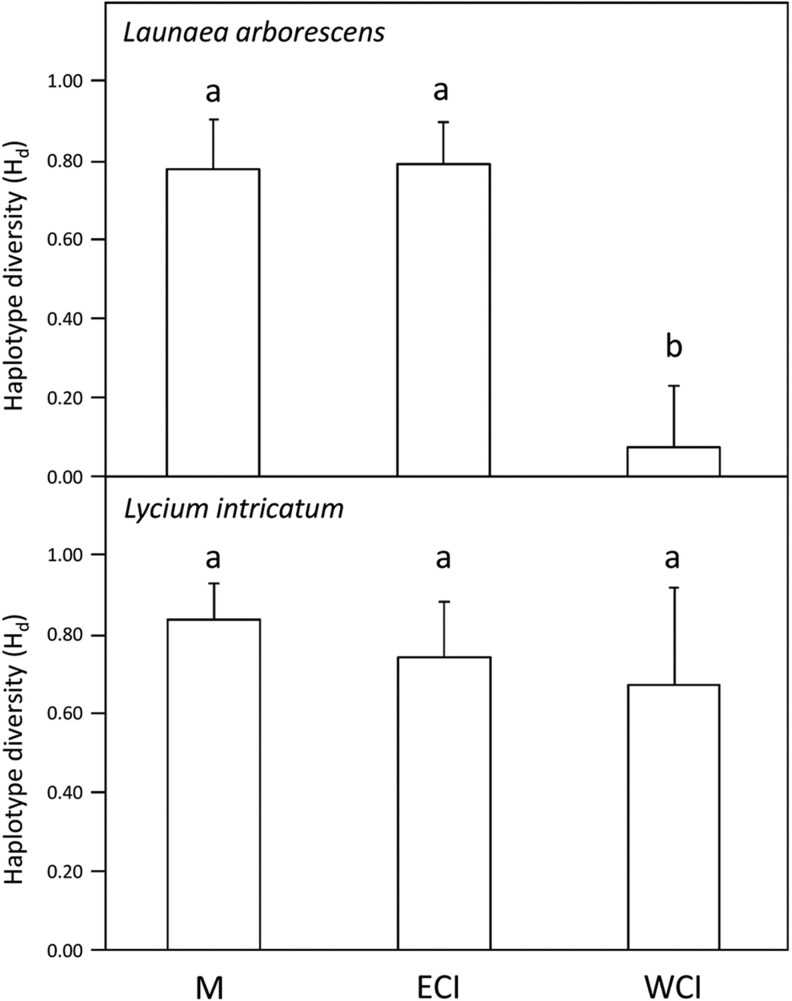
Mean (±SD) levels of haplotype diversity in populations of *Launaea arborescens* and *Lycium intricatum* compared among geographical regions (M = mainland, ECI = easternmost Canarian islands, WCI = western Canarian islands). Different letters above the bars indicate significant differences in haplotype diversity among groups.

Kruskal–Wallis tests revealed significant differences in haplotype diversity among geographical groups in *Launaea* (*χ*^2^ = 9.07, *P* = .011), but did not find significant differences among groups for this variable in *Lycium* (*χ*^2^ = 0.49, *P* = .782). The Dunn *post hoc* test showed that mainland and ECI populations displayed similar levels of haplotype diversity in *Launaea*, but these were significantly lower in WCI populations (Fig. 3).

### Inference of colonization and gene flow patterns based on coalescent approaches

According to the results of DIYABC-RF, the best-supported scenario of colonization was the stepping-stone model (scenario 1: sequential differentiation from M to WCI), with 49% of the votes in *Launaea*, and 51% in *Lycium* (Supplementary Table S3). However, mainland back-colonization (scenario 3) also received substantial support in both species (41% of votes in *Launaea*, and 45% in *Lycium*), which may explain the moderate posterior probability found for scenario 1 (0.538 in *Launaea* and 0.510 in *Lycium*; Supplementary Table S3). This result was consistent with the projection of data on the first two LDA axes, since the training data for both scenario 1 and scenario 3 largely overlapped and were similarly centred around the observed data (Supplementary Figure S6). Parameter estimates for scenario 1 suggested that divergence between M and ECI groups in *Launaea* may have started 2024 generations ago (303–5506; 5%–95% quantiles, respectively; Table 2) and 1961 (287–5520) generations ago in *Lycium*, whereas divergence between ECI and WCI groups may have started 1905 (152–5487) generations ago in *Launaea* and 194 generations ago (14–508) in *Lycium* (Table 2). For scenario 3, back-colonization of mainland from ECI may have started 1806 generations ago (179–4908) in *L. arborescens* and 868 generations ago (324–1527) in *L. intricatum* (Table 2).

**Table 2:** Parameter estimates for divergence times between mainland and easternmost Canarian island populations (*t*_2_) and between easternmost and western Canarian island populations (*t*_1_) for *Launaea arborescens* and *Lycium intricatum* following the coalescent approached implemented in DIYABC-RF.

Species	Scenario	Parameter	Estimate (generations)	Median	5% quantile	95% quantile	Prior error	Posterior error
*L. arborescens*	1	*t*_2_	2024	1418	303	5506	0.46	1.73
*L. arborescens*	1	*t*_1_	1905	1438	152	5487	1.99	2.13
*L. intricatum*	1	*t*_2_	1961	1420	287	5520	0.45	2.41
*L. intricatum*	1	*t*_1_	194	127	14	508	1.73	4.81
*L. arborescens*	3	*t*_1_	1806	1343	179	4908	1.82	2.16
*L. intricatum*	3	*t*_1_	868	813	324	1527	1.79	2.02

Parameters are estimated for the two most-likely scenarios (1 and 3; see text for details). Error rates are based on the normalized mean absolute error.

**Figure 4:**
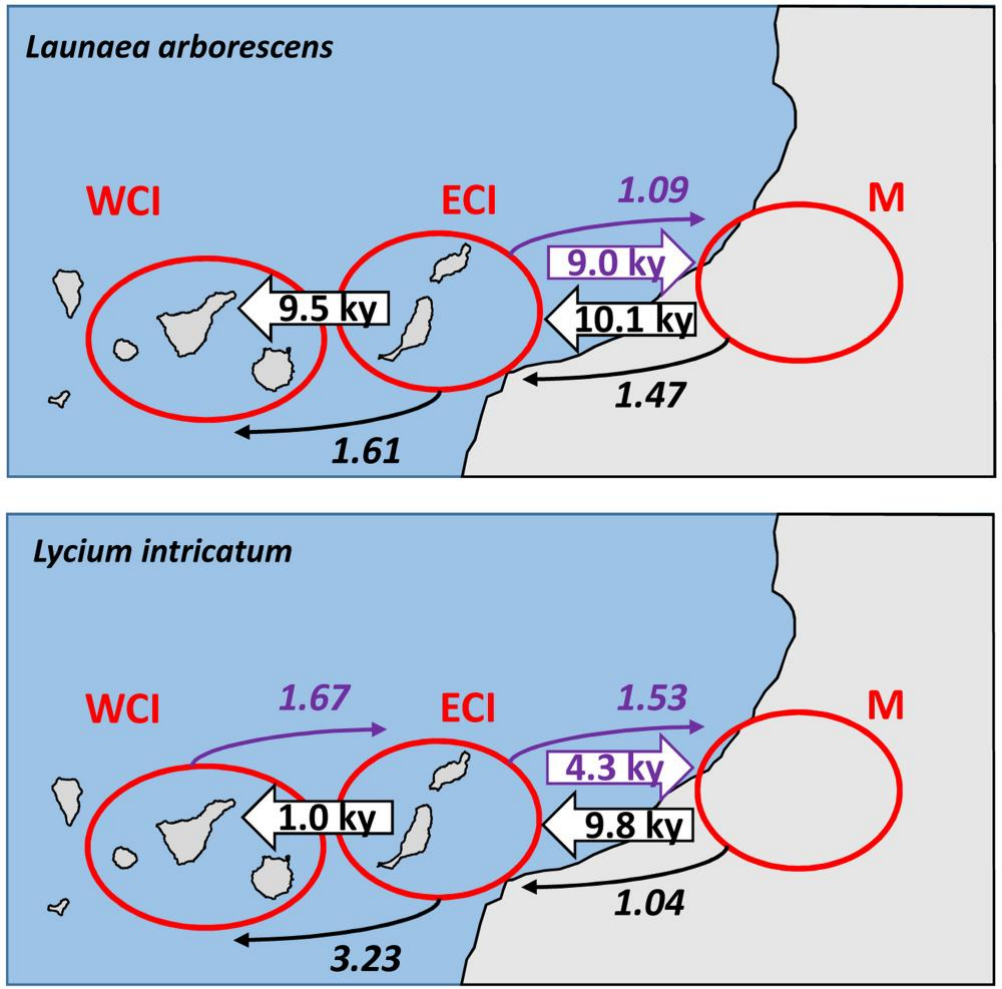
Inferred spatio-temporal patterns of colonization and gene flow in the two study species. Numbers inside arrows indicate estimated divergence times (in thousands of years) between main geographical groups (M = mainland, ECI = easternmost Canarian islands, WCI = western Canarian islands) according to the two, most-likely scenarios inferred from DIYABC-RF (black numbers = scenario 1 and purple numbers = scenario 3; see Results for details). Thin black arrows indicate the direction of gene flow and number in italics above show migration rates (number of migrants per generation) between the three geographical groups as inferred from MIGRATE.

The results from MIGRATE supported that model 3 (bidirectional gene flow between M and ECI + directional gene flow from ECI to WCI) was the most likely for *Launaea*, and model 4 (bidirectional gene flow between M and ECI + bidirectional gene flow between ECI and WCI) for *Lycium* (Supplementary Table S4). Gene flow was inferred to be particularly high between the WCI and the ECI in *Lycium* (ECI → WCI, *N_m_* = 3.23; WCI → ECI, *N_m_* = 1.67), although the inferred best models for each species detected significant levels of gene flow from the ECI to M (*N_m_* = 1.09 in *Launaea* and *N_m_* = 1.53 in *Lycium*; Table 3, Fig. 4).

**Table 3:** Parameter estimates (mean and 95% confidence intervals) based on the model supported for each study species and considering three population groups (mainland = 1, easternmost Canarian islands = 2, western Canarian islands = 3) as inferred from the coalescent approached implemented in MIGRATE.

Parameter	*Launaea*			*Lycium*		
	Mean	95% CI	ESS	Mean	95% CI	ESS
Θ_1_	0.0018	0.0007–0.0031	3223	0.0023	0.0001–0.0044	2133
Θ_2_	0.0020	0.0009–0.0033	2537	0.0031	0.0014–0.0034	2538
Θ_3_	0.0032	0.0019–0.0045	2512	0.0046	0.0035–0.0070	2470
*M*_1→2_	736.7	690.7–970.2	6650	334.4	210.1–474.2	4366
*M*_2→1_	605.3	598.5–854.7	4467	664.2	506.0–999.3	7553
*M*_2→3_	503.0	724.7–976.0	5121	702.8	567.3–999.3	4831
*M*_3→2_	na	na	na	538.8	420.8–962.7	5635

Θ = effective population size; *M* = immigration rate between groups; ESS = effective sample size; na = not applicable.

### Literature survey: levels of haplotype diversity on the easternmost Canarian Islands

Our survey retrieved 12 studies reporting levels of haplotype diversity in Canarian species with widespread distributions within the archipelago (Table 4). The levels of haplotype diversity reported across studies on ECI were not correlated with the number of sequenced individuals (*N*_ECI_; *r* = 0.36, *P* = .30, *N* = 12) or genetic sampling (pDNA; *r* = −0.05, *P* = .87, *N* = 12), which suggests that patterns emerging from these data should not be consistently affected by biases related to differences in sampling effort among studies.

**Table 4:** Haplotype diversity found in molecular studies considering populations on the easternmost Canarian islands (Lanzarote and Fuerteventura) in species with widespread distributions within the archipelago.

Species	Distrib.	pDNA	*N*_ECI_	*H*_ECI_/*H*_ALL_	Hd_ECI_	Ref.
*Astragalus edulis*	NNE	2092 (3)	40	1/1	0	Bobo-Pinilla *et al.* (2018)
*Astydamia latifolia*	NNE	1497 (2)	13	1/4	0	Coello *et al*. (2024a)
*Bituminaria bituminosa*	NNE	2547 (3)	36	5/12	0.22	García-Verdugo *et al.* (2021)
*Euphorbia regis-jubae*	NNE	1996 (4)	12	1/3	0	Sun *et al.* (2016)
*Euphorbia canariensis*	END	1107 (2)	14	2/10	0.13	Coello *et al*. (2024b)
*Kleinia neriifolia*	END	2527 (3)	20	3/17	0.65	García-Verdugo *et al.* (2019a, 2019b)
*Launaea arborescens*	NNE	1539 (3)	36	12/13	0.80	This study
*Lycium intricatum*	NNE	2339 (3)	40	9/12	0.75	This study
*Olea cerasiformis*	END	3282 (4)	12	1/7	0	García-Verdugo *et al.* (2010)
*Periploca laevigata*	END	2782 (3)	15	3/18	0.20	García-Verdugo *et al.* (2017)
*Rumex lunaria*	END	4809 (4)	25	2/30	0.38	González Carracedo *et al.* (2024)
*Scrophularia arguta*	NNE	1142 (2)	10	3/6	0.60	Valtueña *et al.* (2020)

Distribution of each plant species is classified as END (endemic to the Canary Islands) or NNE (native nonendemic). Sampling effort of each study in terms of genetic (pDNA = number of plastid DNA nucleotides sequenced, with number of plastid regions considered in parentheses) and within-population (*N*_ECI_ = number of individuals sequenced on the easternmost islands) representation is detailed. Levels of genetic diversity in the easternmost islands are expressed with two variables: the proportion of haplotypes found on the easternmost islands with regard to the total number of haplotypes found across the Canary Islands (*H*_ECI_/*H*_ALL_) and haplotype diversity (Hd_ECI_, see Materials and methods for calculations).

Overall, we found contrasting levels of haplotype diversity on ECI between our focal species and the studies performed in other Canarian plant species thus far. Excluding the results of the present study, ECI populations displayed, on average, 25% of the haplotypes found across the archipelago (Table 4). Mean haplotype diversity on ECI (Hd_ECI_) across studies was 0.22, with endemics (*H_d_* = 0.27 ± 0.25 SD, *N* = 5) showing a slightly higher value than native nonendemics (*H_d_* = 0.16 ± 0.26 SD, *N* = 5) (Table 4). In contrast, ECI populations in our study species displayed 75% (*Lycium*) and 92% (*Launaea*) of the haplotypes found across the entire archipelago, and both of them displayed the highest values of haplotype diversity reported so far for any other plant species sampled on these islands (*H*_dECI_ = 0.75 in *Lycium* and *H*_dECI_ = 0.80 in *Launaea*; Table 4).

## Discussion

### Similar spatio-temporal patterns in two native non-endemic Canarian species

Our study has revealed some common biogeographical patterns in two key plant components of the lowland, xerophytic habitats of the Canarian archipelago. All of the results pointed towards a scenario of archipelago colonization mediated by extensive rates of gene flow across islands and between mainland and island areas. Our results thus show that native non-endemics in this insular system can challenge some of the classic expectations in island biogeography, such as the progression rule as a model of inter-island dispersal (Shaw and Gillespie 2016), the strong genetic isolation driven by oceanic barriers (Franks 2010) or the low levels of genetic variation on island populations (Frankham 1997). While the emergent patterns in *Launaea* and *Lycium* are related to lowland island habitats, these may have been mirrored by other taxa as a first step for subsequent intra-island colonization and speciation (White *et al.* 2020, Albaladejo *et al.* 2021).

One of the similarities between our focal NNE concerns the spatial distribution of genetic diversity. Thus, both species displayed several haplotypes that were private to the Canarian archipelago (Fig. 2). Although we cannot rule out the possibility that a more extensive sampling could reveal a higher degree of haplotype sharing between island and mainland populations, a well-supported pattern derived from our data is that some of the island haplotypes have been particularly successful in the colonization of the archipelago, for instance LA-15 (white haplotype in Fig. 2) in *Launaea*, or LI-6 (black haplotype) and LI-7 (yellow haplotype) in *Lycium* (Fig. 2). The peripheral position of these haplotypes in the median joining networks (Supplementary Figure S3) points towards a relatively recent origin (i.e. recent divergence from mainland haplotypes). Conversely, mainland haplotypes that are derived from island haplotypes in peripheral positions of the network (e.g. LI-5, LA-11) also suggest the scenario of recent back-colonization of the continent from the islands supported by both coalescent models (Supplementary Table S3 and Supplementary Table S4).

For both species, our analyses suggested that colonization of the Canarian archipelago may have started in relatively recent times (around 10 000 years ago under scenario 1, considering an estimated generation time of 5 years for each species; Fig. 4). This result, and the high rates of gene flow shown by both species across the study system (Table 1; Fig. 4), indicate that inter-island colonization can be a relatively rapid process. Thus, our study reinforces the idea that taxa currently listed as ‘native, non-endemic’ in the Canary Islands represent lineages at contrasting evolutionary stages. While some studies suggest that native non-endemic species display private haplotypic diversity across their entire island distribution (including the most isolated, westernmost islands of La Palma and El Hierro) as a likely result of older colonization times and limited colonization ability (Valtueña *et al.* 2020, Coello *et al.* 2021), our focal species apparently represent another scenario in which haplotype diversity concentrates on the islands closest to the mainland source area as a likely result of more recent (and frequent) island colonization events. While the spatial distribution of genetic diversity is remarkably different among the Canarian native non-endemics analysed thus far, further studies using complementary molecular markers would help clarify the temporal scenario depicted in our study, where extensive gene flow increases uncertainty in model choice (Table 2).

Both *Lycium* and *Launaea* showed a pattern that was only partly compatible with a stepping-stone model of colonization, since historical gene flow was also inferred to be particularly extensive from island to mainland areas (Supplementary Table S3; Fig. 4). Such a common finding supports the idea that colonization in near shore islands of species with high colonization ability can be hardly represented by a single theoretical model. The pattern of recurrent gene flow between island and mainland areas detected in our study is in agreement with the postulates of the syngameon hypothesis (Caujapé-Castells *et al.* 2017), and ultimately accounts for the extraordinary levels of genetic diversity found on the easternmost island populations of both species (Table 4). Their high levels of haplotype diversity (Fig. 3) are thus in agreement with the idea that island species with strong colonization ability (i.e. showing widespread island distributions), do not necessarily display limited genetic variation (García-Verdugo *et al.* 2015). Notably, the highest levels of haplotype diversity reported on the easternmost islands before the present study were those of the Canarian endemic *Kleinia neriifolia*, and of *Scrophularia arguta*, a native non-endemic taxon (Table 4). In both cases, phylogeographical analyses suggested that moderate levels of island genetic diversity were likely due to multiple events of dispersal, either from the continent in *Scrophularia* (Valtueña *et al.* 2020) or from the central islands in the case of *Kleinia* (García-Verdugo *et al.* 2019a, García-Verdugo et al. 2019b, Rincón Barrado *et al.* 2024). The key role of gene flow in the spatial distribution of genetic diversity is clearly exemplified in *Launaea* and *Lycium*, since our results showed that easternmost islands represent *de facto* a genetic continuum of the neighbouring mainland area (Supplementary Figure S5), even though populations are geographically separated by oceanic barriers of more than 100 km.

### Contrasting patterns of gene flow between native, non-endemic species

Despite the similar patterns outlined above, our results also indicated particular differences in the biogeographical signature of each focal species. The most remarkable one is that *Lycium* displayed higher rates of gene flow than those inferred for *Launaea*, which translates into island haplotypes showing wider distribution ranges (Fig. 2; Supplementary Figure S4), and negligible differentiation among geographical groups (Table 1).

Notwithstanding the possible effects of ornithochory (which typically promotes high rates of inter-island dispersal; García-Verdugo *et al.* 2015, Arjona *et al.* 2018), we hypothesize that secondary seed dispersal by predatory birds may have been the main force accounting for the extensive levels of gene flow detected in *Lycium*. Thus, among the seeds dispersed by shrikes (*Lanius meridionalis koenigi*, syn: *Lanius excubitor koenigi*), Padilla *et al.* (2012) found that 70% of them belonged to fleshy-fruited species that typically occur in open, coastal habitats, with *Lycium intricatum* being one of the most frequent. The pattern of recurrent gene flow between mainland and island areas and across the archipelago has been also reported among shrike populations, which suggests phylogeographical congruence between the potential dispersal vector and the frequently dispersed plant species (Padilla *et al.* 2015). The hypothesis of shrikes as an important dispersal vector would help explain why *Lycium* has not been established yet on the westernmost Canarian islands of El Hierro and La Palma, where this bird species is not known to occur (Biota database; https://www.biodiversidadcanarias.es/biota/especie/V00058). Further research would be needed to examine whether secondary seed dispersal is the process explaining the low levels of genetic structure detected in our study (e.g. by performing DNA analyses of *Lycium* seeds extracted from shrike pellets).

## Conclusions

Native non-endemic taxa represent a major proportion of island biodiversity (Schrader *et al.* 2024). In the Canary Islands, one of the most studied archipelagos in the world, c. 1000 NNE plants are recognized to date (Beierkuhnlein *et al.* 2021), but very few studies have addressed the biogeographical patterns associated with the process of island colonization in this type of taxa. As posited in Caujapé-Castells *et al.* (2017), the study of widespread NNE is likely to enhance our understanding of the first stages of plant diversification on islands, as most of these lineages are very recent island colonizers, and likely have been less affected by extinction.

The present study has focused on NNE with widespread distributions within the Canarian archipelago, but species with narrower distributions in this, or other, archipelagos could provide a complementary view. Phylogeographical studies specifically targeting island distributions of NNE are much needed to gain further insight into the otherwise limited genetic pattern revealed by phylogenetic analysis (Martín-Hernanz *et al.* 2023). If we are to understand the patterns associated with island biodiversity at all hierarchical levels, we do encourage to expand current trends in island biogeography to this type of taxa. Even though our study is based on the information extracted from small fragments of plastid DNA, we found sufficient genetic variation to describe robust patterns across the island distribution of our focal plant species. It is likely that larger genetic sampling (plastome analyses) would be needed in other native non-endemic species showing more limited genetic variation while the use of genetic (nuclear) markers could provide complementary information in future studies.

At the regional scale, our results reinforce the idea that NNE represent a major biogeographical component of the Macaronesian region, since they portray particular spatio-temporal patterns of insular colonization (Fernández-Palacios *et al.* 2024). At a broader scale, phylogeographical studies on NNE are needed to successfully integrate genetic patterns into the existing body of island biogeography theory (Overcast *et al.* 2023).

## Supplementary Material

plag021_Supplementary_Data

## Data Availability

The DNA sequences generated in this study are deposited in the GenBank database under the accession numbers PV554281–PV554643 (for *Lycium intricatum*) and GenBank accessions PV255023–PV255258 (for *Launaea arborescens*).
