# Supplementary material for: Taking the common into account: phylogeographical patterns of native, non-endemic plant species of the Canary Islands
Source: AoB Plants. 2026 May 20;18(3):plag021. doi: 10.1093/aobpla/plag021 (PMC13394810; doi:10.1093/aobpla/plag021)

SUPPLEMENTARY INFORMATION

Table S1. List of populations sampled for each species in this study, sample size (N), coordinates and haplotypes for each population. Numbers in parentheses indicate individuals displaying the same haplotype within a given population.

**Species**	**Pop**	**N**	**Code**	**Region/island**	**Coordinates**	**Haplotype(s)**
L. arborescens	Anza	6	ANZ	mainland	30°28'02"N 9°39'41"W	LA-1 (1), LA-5 (3), LA-12 (1), LA-17 (1)
L. arborescens	Ida Ougnidif	5	IDA	mainland	29°51'59"N 9°00'49"W	LA-3 (2), LA-6 (1), LA-14 (1), LA-16 (1)
L. arborescens	Aït Bou Nouh	6	NOU	mainland	29°30'45"N 8°54'18"W	LA-2 (1), LA-8 (1), LA-9 (3), LA-14 (1)
L. arborescens	Legzira	6	LEG	mainland	29°26'53"N 10°06'42"W	LA-5 (1), LA-6 (1), LA-10 (4)
L. arborescens	Famara	6	FAM	Lanzarote	29°07'18"N 13°31'54"W	LA-2 (1), LA-10 (2), LA-11 (1), LA-14 (1), LA-17 (1)
L. arborescens	Tahiche	6	TAH	Lanzarote	29°01'00"N 13°33'05"W	LA-2 (1), LA-5 (1), LA-13 (3), LA-18 (1)
L. arborescens	Femés	6	FEM	Lanzarote	28°55'06"N 13°46'42"W	LA-1 (1), LA-2 (1), LA-7 (1), LA-14 (3)
L. arborescens	Montaña Tindaya	6	TIN	Fuerteventura	28°35'52"N 13°58'43"W	LA-7 (2), LA-14 (2), LA-15 (2)
L. arborescens	Risco Carnicero	6	CRN	Fuerteventura	28°22'46"N 14°04'39"W	LA-2 (1), LA-4 (1), LA-14 (4)
L. arborescens	Jandía	6	JAN	Fuerteventura	28°06'20"N 14°23'26"W	LA-2 (1), LA-14 (3), LA-18 (1), LA-19 (1)
L. arborescens	Tafira	6	TAF	Gran Canaria	28°04'25"N 15°26'43"W	LA-15 (6)
L. arborescens	Montaña Agüimes	6	AGU	Gran Canaria	27°53'50"N 15°26'18"W	LA-15 (6)
L. arborescens	Andén Verde	6	ANV	Gran Canaria	27°05'03"N 15°42'15"W	LA-14 (1), LA-15 (5)
L. arborescens	Arguineguín	6	BAR	Gran Canaria	27°49'22"N 15°39'48"W	LA-15 (6)
L. arborescens	Anaga	6	ANA	Tenerife	28°30'59"N 16°10'32"W	LA-15 (6)
L. arborescens	Fasnia	5	FAS	Tenerife	28°13'52"N 16°25'51"W	LA-14 (1), LA-15 (4)
L. arborescens	Punta de Rasca	6	RAS	Tenerife	28°00'59"N 16°41'49"W	LA-15 (6)
L. arborescens	Teno-El Rayo	6	TEN	Tenerife	28°23'32"N 16°49'53"W	LA-15 (6)

L. arborescens	Aluce	6	ALU	La Gomera	28°07'27"N 17°06'48"W	LA-15 (6)
L. arborescens	Bco. Argaga	6	ARG	La Gomera	28°04'55"N 17°19'15"W	LA-15 (6)
L. intricatum	Anza	6	ANZ	mainland	30°28'02"N 9°39'41"W	LI-1 (2), LI-2 (1)- LI-3 (1), LI-4 (2)
L. intricatum	Agadir	6	AGA	mainland	30°21'00"N 9°27'55"W	LI-1 (1), LI-2 (1), LI-3 (2), LI-4 (2)
L. intricatum	Legzira	5	LEG	mainland	29°26'53"N 10°06'42"W	LI-1 (3), LI-13 (1), LI-14 (1)
L. intricatum	Fort Bou-Jerif	5	FBJ	mainland	29°05'13"N 10°19'54"W	LI-1 (1), LI-3 (1), LI-14 (2), LI-15 (1)
L. intricatum	Famara	6	FAM	Lanzarote	29°07'18"N 13°31'54"W	LI-1 (1), LI-10 (3), LI-15 (2)
L. intricatum	Tahiche	5	TAH	Lanzarote	29°01'00"N 13°33'05"W	LI-1 (1), LI-7 (3), LI-10 (1)
L. intricatum	Femés	6	FEM	Lanzarote	28°55'06"N 13°46'42"W	LI-10 (4), LI-11 (1), LI-17 (1)
L. intricatum	Montaña Tindaya	6	TIN	Fuerteventura	28°35'52"N 13°58'43"W	LI-1 (4), LI-15 (1), LI-17 (1)
L. intricatum	Risco Carnicero	6	CRN	Fuerteventura	28°22'46"N 14°04'39"W	LI-1 (2), LI-8 (1), LI-10 (1), LI-11 (1), LI-17 (1)
L. intricatum	Montaña Cardón	6	CAR	Fuerteventura	28°15'46"N 14°07'57"W	LI-6 (1), LI-7 (2), LI-10 (1), LI-11 (1), LI-12 (1)
L. intricatum	Jandía	5	JAN	Fuerteventura	28°06'20"N 14°23'26"W	LI-6 (3), LI-10 (1), LI-15 (1)
L. intricatum	Tinoca	6	TNO	Gran Canaria	28°08'17"N 15°29'19"W	LI-1 (1), LI-7 (2), LI-10 (2), LI-11 (1)
L. intricatum	Montaña Agüimes	6	AGU	Gran Canaria	27°53'50"N 15°26'18"W	LI-1 (1), LI-6 (1), LI-10 (1), LI-16 (1), LI-17 (2)
L. intricatum	Andén Verde	5	ANV	Gran Canaria	27°05'03"N 15°42'15"W	LI-1 (1), LI-6 (1), LI-8 (1), LI-9 (1), LI-11 (1)
L. intricatum	Arguineguín	6	BAR	Gran Canaria	27°49'22"N 15°39'48"W	LI-1 (1), LI-7 (2), LI-10 (2), LI-17 (1)
L. intricatum	Almáciga	6	ALM	Tenerife	28°34'16"N 16°11'29"W	LI-1 (2), LI-7 (4)
L. intricatum	Fasnia	6	FAS	Tenerife	28°13'52"N 16°25'51"W	LI-1 (1), LI-8 (5)
L. intricatum	Punta de Rasca	6	RAS	Tenerife	28°00'59"N 16°41'49"W	LI-6 (5), LI-17 (1)
L. intricatum	Teno-Buenavista	6	TEN	Tenerife	28°21'40"N 16°54'02"W	LI-1 (3), LI-15 (3)
L. intricatum	Bco. de Cocos	6	COC	La Gomera	28°02'26"N 17°13'14"W	LI-5 (2), LI-7 (3), LI-10 (1)
L. intricatum	Alojera	6	ALO	La Gomera	28°09'32"N 17°19'17"W	LI-1 (4), LI-17 (2)

Table S2. SAMOVA results based on plastid DNA sequence information for the study species (*Launaea arborescens* and *Lycium intricatum*) considering an increased number of genetically homogeneous groups (K values from 2 to 4).

	Launaea				*Lycium*			
	**df**	**SS**	**VC**	**%Var**	**df**	**SS**	**VC**	**%Var**
**K = 2**								
Among areas	1	691.7	11.61	75.3	1	16.6	1.17	30.5
Among pops (areas)	18	118.3	0.56	3.6	19	111.4	0.67	17.4
Within pops	98	319.0	3.25	21.1	100	200.0	2.00	52.1
**K = 3**								
Among areas	2	756.9	10.25	76.0	2	46.9	1.20	32.9
Among pops (areas)	17	53.1	0.02	0.1	18	90.2	0.52	13.4
Within pops	98	319.0	3.25	23.9	100	200.0	2.00	52.7
**K = 4**								
Among areas	3	764.9	10.0	75.8	3	45.0	1.16	32.7
Among pops (areas)	16	45.1	0.06	0.1	17	84.6	0.60	14.9
Within pops	98	319.0	3.25	24.1	100	200.0	2.00	52.4

df = degrees of freedom; SS = sum of squares; VC = variance component; %Var = percentage of explained variance

Table S3. Summary of the results for model choice considering the three scenarios of genetic differentiation tested in *Launaea arborescens* and *Lycium intricatum* in the framework implemented in DIYABC-RF based on 10,000 decision trees. See Materials and Methods for details on each scenario.

Dataset	votes scenario 1	votes scenario 2	votes scenario 3	selected scenario	posterior probability
Launaea	4854	960	4186	1	0.538
Lycium	5071	481	4448	1	0.510

Table S4. Posterior probabilities for each of the four models of historical gene flow tested in *Launaea arborescens* and *Lycium intricatum* with the coalescent approach implemented in MIGRATE. Models with the smallest posterior probabilities (in bold) were selected as the most likely. See Materials and Methods for details on each model.

	**ln(Prob(D|Model))**			
**Dataset**	**Model 1**	**Model 2**	**Model 3**	**Model 4**
Launaea	-2737.9	-2071.1	**-1996.9**	-2081.1
Lycium	-4242.6	-3227.4	-3235.1	**-3115.5**

Figure S1. Phylogenetic reconstruction based on Bayesian inference using the plastid sequence dataset of each species. Stars represent the nodes that received high statistical support (PP > 0.9) and colours of each label represent sample origin (island or mainland).

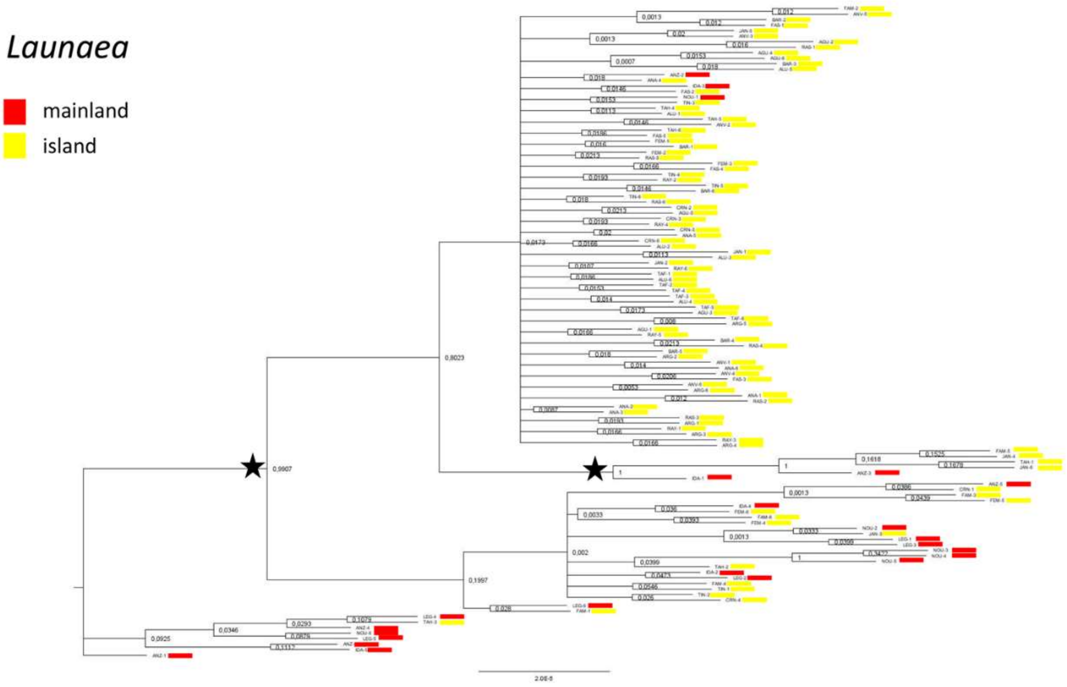

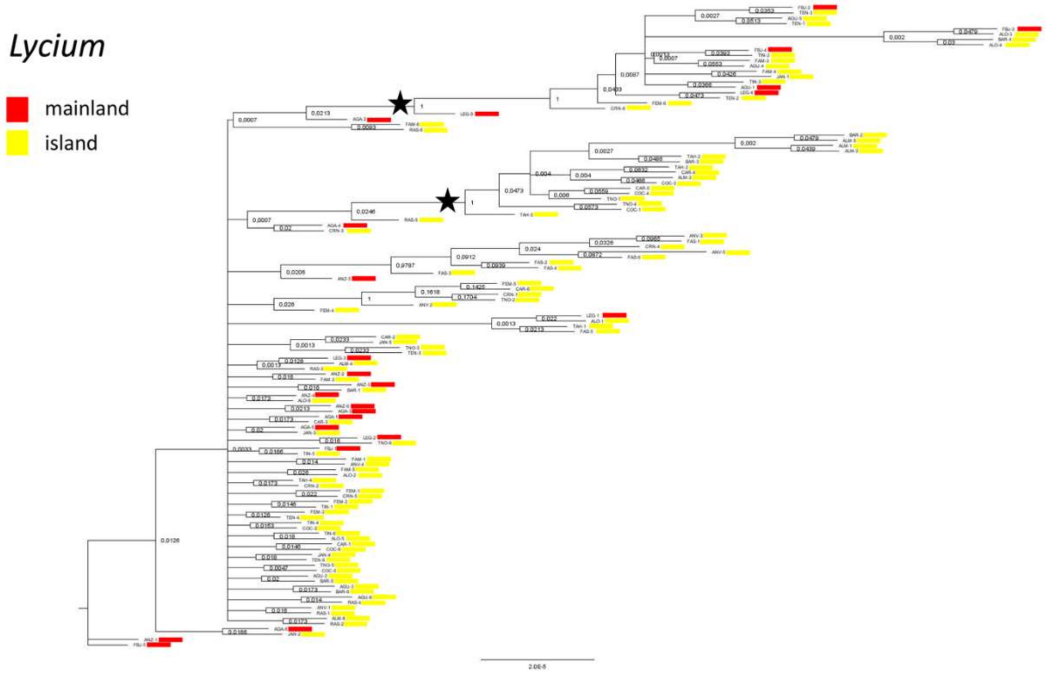

Figure S2. Models tested for analyzing the best supported pattern of gene flow in the two study species as inferred from the coalescent approach implemented in MIGRATE. The most simple model (Model 1) represents a stepping-stone model of differentiation with complete isolation (i.e. lack of gene flow) between geographical groups (M = mainland, ECI = easternmost Canarian islands, WCI = western Canarian islands). Blue arrows represent unidirectional gene flow between geographical groups, whereas green arrows represent bidirectional gene flow between them.

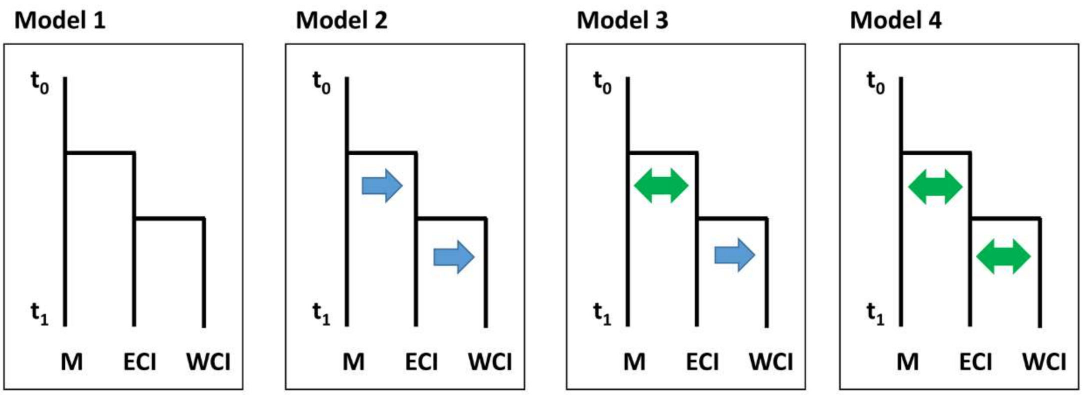

Figure S3. Haplotype networks based on the analyses of plastid DNA sequences in *Launaea arborescens* and *Lycium intricatum* using the median joining algorithm implemented in NETWORK (closed marks = indels; open marks = substitutions).

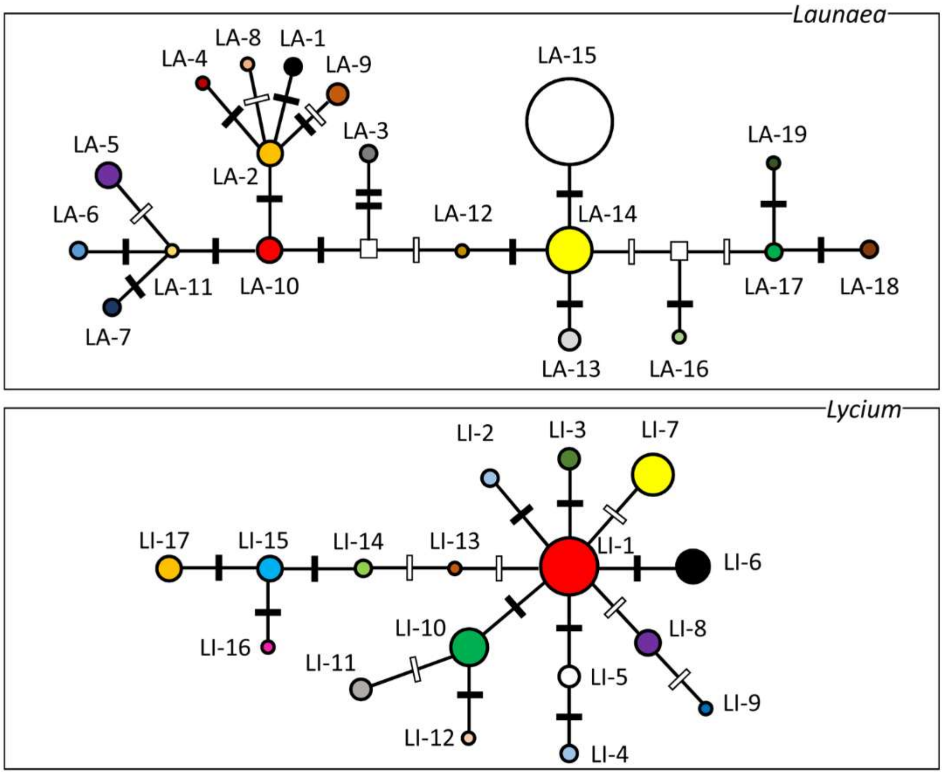

Figure S4. Distribution range (occurrence on different islands) of the haplotypes found in *Launaea arborescens* and *Lycium intricatum* within the Canarian archipelago. Rare haplotypes (found in one individual only) are not considered.

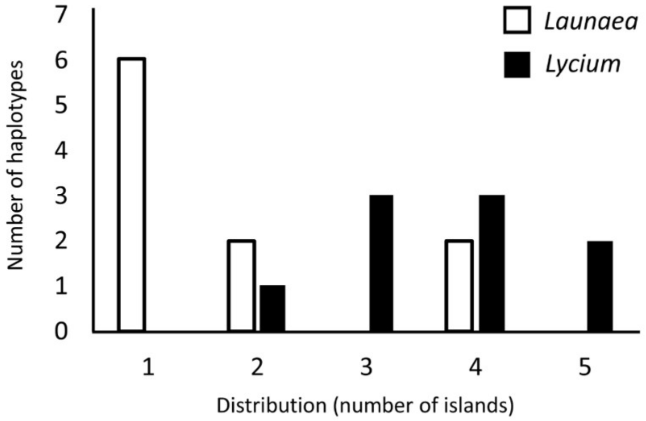

Figure S5. SAMOVA output showing genetically homogeneous groups (dots in each map with same color) considering K = 2 for the *Launaea arborescens* and *Lycium intricatum* populations sampled in this study.

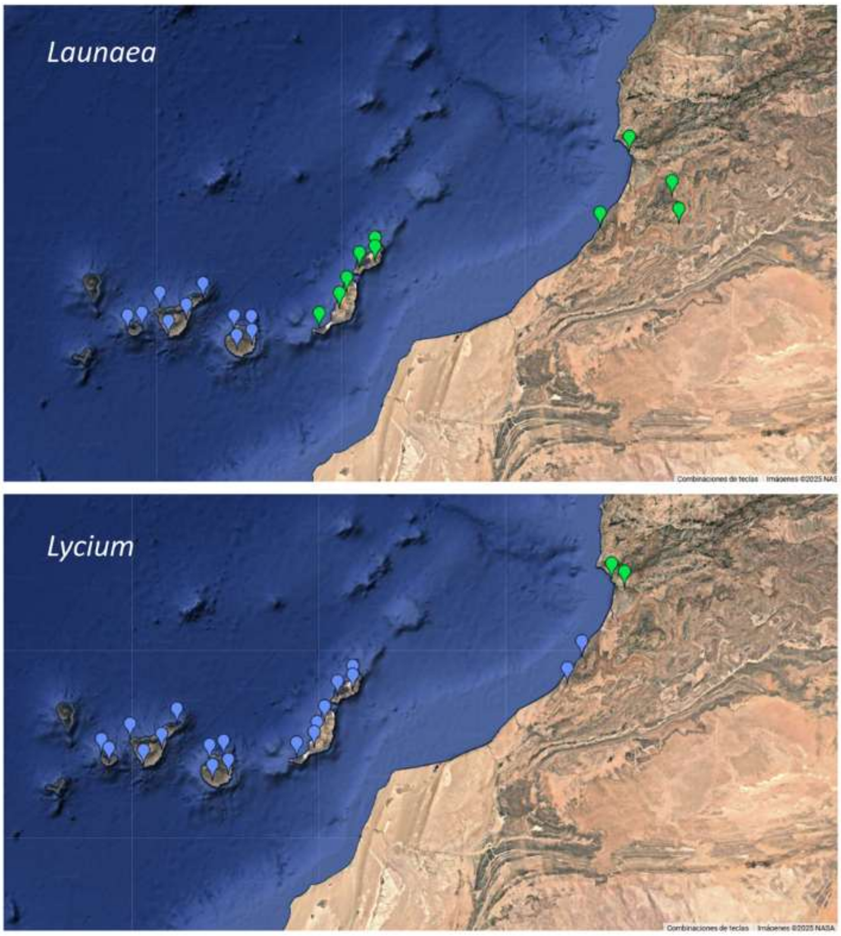

Figure S6. Plots of the Linear Discriminant Analysis showing the projection of training and observed datasets onto the first two axes obtained from the coalescent analysis implemented in DIYABC-RF.

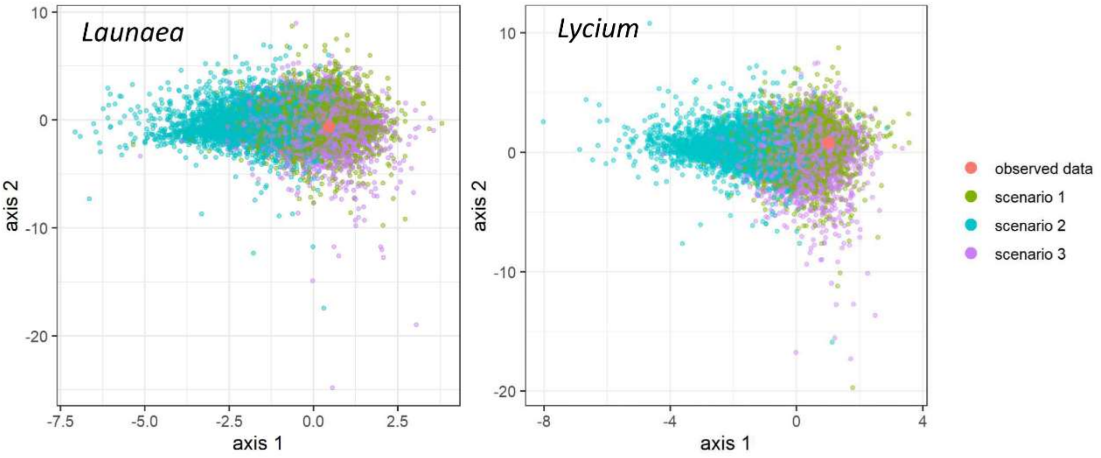

Supplement: plag021_Supplementary_Data [file plag021_supplementary_data.pdf]
